# Enhancing nasal irrigation adherence through a multimodal health communication strategy: a phased improvement study

**DOI:** 10.1038/s41598-026-67975-z

**Published:** 2026-09-02

**Authors:** Lok-Yee Joyce Li, Sheng-Yu Wu, Cheng-Yu Tsai, Arnab Majumdar, Jeffrey Yang, Chien-Ling Su, Cheng-Jung Wu

**Affiliations:** 1 Department of Environmental and Occupational Medicine, National Taiwan University Hospital, Hsin-Chu Branch, Hsinchu, Taiwan; 2 Department of Otolaryngology, School of Medicine, College of Medicine, Taipei Medical University, Taipei, Taiwan; 3 Department of Industrial Engineering and Industrial Management, National Tsing Hua University, Hsinchu, Taiwan; 4 Department of Civil and Environmental Engineering, Imperial College London, London, UK; 5 School of Respiratory Therapy, College of Medicine, Taipei Medical University, Taipei, Taiwan; 6 TMU Research Center of Artificial Intelligence in Medicine and Health, Taipei Medical University, Taipei, Taiwan; 7 Department of Biology, University of California, Riverside, CA USA; 8 Department of Otolaryngology, Shuang Ho Hospital, Taipei Medical University, Taipei, Taiwan; 9 Biomedical Science and Engineering, National Yang Ming Chiao Tung University, Hsinchu, Taiwan; 10 Allergy and Rhinology, Quality HealthCare Center, Taoyuan, Taiwan

**Keywords:** Patient education, Health communication, Adherence, Allergic rhinitis, Nasal irrigation, Quality improvement, Counseling, Patient-centered care, Diseases, Health care, Medical research

## Abstract

The adherence of chronic rhinitis patients to self-administered treatments is generally very low, which often presents a significant challenge in healthcare. The adherence of chronic rhinitis patients to regular nasal irrigation hygiene care is even lower. Therefore, in this study, we designed, implemented, and evaluated a patient-centered Multimodal Health Communication Strategy intervention, that aimed to improve the adherence to nasal irrigation of adult patients with allergic rhinitis or chronic hypertrophic rhinitis. This was a healthcare quality improvement project conducted from January to July 2025 in the otolaryngology outpatient department of a medical center. The healthcare quality improvement intervention evolved in two stages. The first-stage intervention introduced a “Multimodal Educational Strategy,” combining visual aids, instructional videos, and one-on-one consultation with standard brief verbal instructions. The second-stage intervention, based on patient feedback from the first stage, further optimized the user experience by providing an ergonomic irrigation device and optionally available Herbal Tea Rinse saline solution. The primary outcome measure was that nasal irrigation adherence significantly increased from a baseline of 11.2% to 45.2% after the first stage (*p* < 0.001), and further increased to 75.0% after the second stage (*p* = 0.013). This represents a 569% relative increase (*p* < 0.001) compared to patients who had not undergone the Multimodal Health Communication Strategy intervention. Finally, patients who received the Multimodal Health Communication Strategy educational intervention showed an improvement in knowledge mastery from 51% to 93.8%, and overall satisfaction also significantly increased. The study concluded that low adherence to nasal irrigation is a problem that can be overcome, rather than an insurmountable communication gap between patients and healthcare providers. Systematic patient-centered education through the Multimodal Health Communication Strategy was associated with an improvement in self-nasal irrigation care adherence.

**Supplementary Information:**

The online version contains supplementary material available at https://doi.org/10.1038/s41598-026-67975-z.

## Introduction

 In Taiwan, allergic rhinitis (AR) and chronic hypertrophic rhinitis (CHR) are highly prevalent upper respiratory diseases in adults. Approximately 26.3% of the population in Taiwan is affected by rhinitis^1^. Chronic rhinitis patients experience persistent symptoms such as nasal congestion and a runny nose. These symptoms can severely impair sleep quality, work efficiency, and overall quality of life^2^. Treatment of these chronic rhinitis conditions often involves long-term medication, including oral drugs and corticosteroid nasal sprays. Although these medications are effective, they often increase the risk of side effects and also raise healthcare costs.

Nasal irrigation is a safe, cost-effective, and medically supported adjunct therapy for rhinitis. The principle of nasal irrigation is to physically wash away allergens and inflammatory mediators from the nasal cavity. Previous studies showed that nasal irrigation can also improve the function of nasal mucosal cilia^3,4^. Medical evidence from Cochrane systematic reviews confirmed that nasal irrigation, in addition to relieving nasal congestion and allergy symptoms, can also reduce patients’ medication usage^5,6^. It has a moderate to high efficacy in treating rhinitis, and the side effects of nasal irrigation are minimal. Other studies indicated a strong correlation between good adherence to nasal irrigation and clinical treatment outcomes in patients (including both adults and children) with rhinitis^7^.

Although nasal irrigation was proven to have significant therapeutic effects, in the real world, there is still a substantial gap in patients’ willingness to use nasal irrigation to treat rhinitis. In clinical practice, physicians often recommend that patients do nasal irrigation. However, patients often don’t follow through properly, which becomes a big challenge in doctor-patient communication^8^. In most hospital outpatient clinics, physicians can only verbally instruct rhinitis patients to perform nasal care at home. We observed that under this minimal doctor-patient communication model, the adherence rate of rhinitis patients to nasal irrigation was only 11.2%. A preliminary analysis showed that this was not due to the lack of effectiveness of nasal irrigation, but because of patients’ inadequate medical education and psychological barriers. Among these, insufficient understanding of the benefits of nasal irrigation accounted for 48.9%, patients’ fear of discomfort from water entering the airway accounted for 30.6%, and some rhinitis patients simply considered nasal irrigation to be bothersome. Therefore, the challenge does not lie in whether the treatment itself is effective enough, but in the effectiveness of health education communication.

The aim of this quality improvement study was to address the gaps in health education communication. Using the Plan-Do-Check-Act (PDCA) framework^9^, we hoped that this quality improvement study could effectively go beyond the traditional, one-way guidance model of ‘information provision.’ Therefore, we were committed to developing and implementing a patient-centered quality improvement plan for a Multimodal Health Communication Strategy, aiming to provide rhinitis patients with the necessary knowledge, skills, and confidence to perform nasal irrigation, enabling them to successfully adopt and maintain nasal irrigation as part of their self-care routine for chronic rhinitis.

## Methods

### Study design and team

This quality improvement study was conducted from January to July 2025 at the otolaryngology outpatient clinic of a university-affiliated medical center. A multidisciplinary team, named the “Nasal Care Circle,” comprising an otolaryngologist, registered nurses, and a patient educator, guided the project using the PDCA method. All methods were performed in accordance with relevant guidelines and regulations. Unlike traditional randomized controlled trials, this study used a practical PDCA framework, making quick clinical adjustments repeatedly based on real-world patient feedback.

### Participants and baseline assessment

Participants of this study were adults who had been diagnosed with allergic rhinitis or chronic hypertrophic rhinitis and who, at the same time, were recommended by a specialist in the ear, nose and throat (ENT) outpatient clinic to perform nasal irrigation. Baseline compliance with nasal irrigation was assessed in 98 rhinitis patients, 62 rhinitis patients participated in the first phase of the study, and 32 rhinitis patients participated in the second phase of the study. The recruitment method was consecutive enrollment from rhinitis patients in the ENT clinic who met the criteria. Exclusion criteria included patients who had recently undergone nasal surgery or those with contraindications to nasal irrigation. As to baseline nasal irrigation compliance, we found that in the baseline survey of the 98 eligible rhinitis patients, only 11 performed home nasal irrigation, meaning baseline compliance was only 11.2%. Key barriers identified through a Pareto analysis were primarily related to knowledge gaps and negative perceptions. Since this quality improvement project was carried out in an ENT clinic with a high patient volume, we couldn’t conduct a continuous study on the same patient using both phase 1 and phase 2 at the same time. So, we used consecutive sampling for independent patients who came in at each phase, rather than a longitudinal follow-up design.

The Multimodal Health Communication Strategy.

Intervention measures were implemented in two different phases, each targeting different areas of patient learning and experience.

### Phase 1 (March–April): the multimodal educational strategy

The purpose of this phase of research was to address cognitive (knowledge) and initial emotional (fear, uncertainty) barriers to nasal irrigation by establishing a structured and supportive learning environment. According to the multimodal **e**ducational strategy, it can enhance understanding of nasal irrigation knowledge and the correct way to perform nasal irrigation. This strategy included Optimized Educational Materials, Standardized Doctor-Led Counseling, and Resource Linkage and Follow-up.

1. Optimized educational materials: for this project, we developed an engaging visual instructional chart that explains the principles of nasal irrigation and the correct steps for nasal irrigation in an easy-to-understand manner. In addition, we also provided a QR code link to a standardized instructional video, allowing rhinitis patients to continue asynchronous learning at home and strengthen their nasal irrigation skills.
2. Standardized doctor-led counseling: all participating physicians received training in health counseling. The physicians conduct a 5-minute one-on-one consultation with rhinitis patients, including on-site demonstrations. In addition, during the consultation, physicians used ‘active listening’ to identify individual obstacles that prevented patients from rinsing their nose (for example, some patients feel like they are choking when performing nasal rinsing). They then addressed these difficulties through personalized doctor-patient communication, thereby building the patients’ trust and their ability to perform nasal rinsing at home on their own^10,11^.
3. Resource linkage and follow-up: after the outpatient visit, patients with rhinitis received a recommended list of nasal irrigators to purchase, and 1 week after the consultation, they received a follow-up call. At this time, assistance was provided to rhinitis patients to resolve any difficulties with nasal irrigation, while also offering emotional support.

### Phase 2 (May–June): experience optimization through patient feedback

Through patient feedback to optimize the experience, phase 1 of the quality improvement study focused on patient hygiene education. During this time, continuous communication between the research team and patients revealed that long-term compliance with nasal irrigation was also closely related to their user experience. Many patients indicated that traditional nasal irrigation devices are difficult to operate and that saline solutions are highly irritating. To directly respond to this patient feedback, the research team added a second phase of the quality improvement study. The purpose of this phase of the study was to optimize the nasal irrigation experience for patients with rhinitis.

1. Ergonomic nasal irrigation device: the research team personally designed a nasal irrigation device that is more ergonomic, easier to grasp, and has better control of the flow direction. The new Ergonomic Irrigation Device also reduces the difficulty of operation and side effects caused by nasal irrigation.
2. Herbal tea rinse saline solution: an optional saline solution infused with mild, soothing herb and tea extracts was offered as an alternative to enhance comfort and create a more-pleasant sensory experience. The solution can effectively reduce the irritation of traditional nasal rinse fluid. This natural nasal irrigation solution is a safety-verified product. During the study, no reports of adverse mucosal events were received.

### Outcome measures

The primary outcome was the adherence rate (%), defined as the proportion of patients self-reporting regular nasal irrigation at home during follow-up calls. Secondary outcomes included knowledge mastery (%), measured by a short questionnaire; symptom severity, assessed using a 10-point visual analog scale (VAS); weekly use of rescue medication; and overall satisfaction, rated on a five-point Likert scale.

### Statistical analysis

Data were analyzed using SPSS (SPSS, Armonk, NY, USA). An overall Chi-squared test was conducted to compare adherence rates across all three independent cohorts. For survey instruments, we have highlighted the high inter-rater reliability (Fleiss’ kappa = 0.85) as evidence of the instrument’s robustness, alongside paired comparisons before and after the intervention.” A *p* value of < 0.05 was considered statistically significant.

### Ethics approval and consent to participate

This study was approved by the Taipei Medical University-Joint Institutional Review Board (TMU-JIRB N202509005). All methods were performed in accordance with the relevant guidelines and regulations. As a quality improvement project utilizing anonymized data from routine clinical practice, the board granted a waiver of the requirement for written informed consent.

## Results

### Primary outcome: improvement in adherence rates

As shown in Table 1, demographic characteristics including age, gender, diagnosis, disease duration, and education level were across the three independent cohorts, with no statistically significant differences observed (all *p* > 0.05).”

This hygiene education measure led to a statistically significant increase in nasal irrigation compliance among rhinitis patients (see Table 2). When patients only received the physician’s verbal instruction to “rinse your nose at home to improve rhinitis,” the baseline nasal irrigation compliance rate was 11.2% (that is, out of 98 rhinitis patients, only 11 rinsed their nose at home). After implementing phase 1 of The Multimodal Educational Strategy, the compliance rate rose to 45.2% (28/62), an increase of 33.8% points (χ²=21.7, *p* < 0.001). Following the phase 2 patient experience optimization measures, the nasal irrigation compliance further increased to 75.0% (24/32), an increase of 29.8% points compared to the previous phase (χ²=6.12, *p* = 0.013). Overall, from the baseline to completion of the two-phase Multimodal Health Communication Strategy, this quality improvement study achieved a 569% relative increase in compliance (*p* < 0.001). An overall comparison across the three cohorts (baseline, phase 1, and phase 2) demonstrated a statistically significant difference in nasal irrigation adherence (overall χ²=50.84, p < 0.001).”

### Secondary outcomes

The Multimodal Educational Strategy also achieved significant improvements in secondary indicators (see Table 3). The proportion of patients demonstrating knowledge mastery increased from 51% at the baseline to 93.8% after receiving the Multimodal Educational Strategy (*p* < 0.01). The average overall satisfaction of patients with their nasal irrigation care plan increased from 3.0 to 4.7 points (on a five-point Likert scale, *p* < 0.01). In terms of clinical performance, patients’ self-reported average nasal symptom score decreased from 8.3 to 3.2 points (*p* < 0.001). The average weekly frequency of medication use among patients with rhinitis also decreased from 5.2 times to 2.1 times (*p* < 0.001).

**Table 1:** Baseline demographic and clinical characteristics across three cohorts.

Characteristics	Baseline (*n* = 98)	Phase 1 (*n* = 62)	Phase 2 (*n* = 32)	*p*-value
**Age (years)**, mean ± SD	42.6 ± 11.8	41.9 ± 12.4	43.2 ± 10.9	0.86^a^
**Gender**, n (%)				0.81^b^
Male	42 (42.9%)	25 (40.3%)	15 (46.9%)	
Female	56 (57.1%)	37 (59.7%)	17 (53.1%)	
**Diagnosis**, n (%)				0.90^b^
Allergic rhinitis	69 (70.4%)	42 (67.7%)	23 (71.9%)	
Chronic hypertrophic rhinitis	29 (29.6%)	20 (32.3%)	9 (28.1%)	
**Disease duration (years)**, mean ± SD	5.8 ± 3.1	5.4 ± 2.8	6.1 ± 3.4	0.48^a^
**Education level**, n (%)				0.96^b^
High school or below	18 (18.4%)	10 (16.1%)	6 (18.8%)	
College/university	62 (63.3%)	40 (64.5%)	19 (59.4%)	
Postgraduate	18 (18.4%)	12 (19.4%)	7 (21.9%)	

Data are presented as n (%) or mean ± SD where appropriate.

^a^ Calculated using one-way ANOVA.

^b^ Calculated using Chi-squared test.

**Table 2:** Phased improvement in nasal irrigation adherence.

Intervention phase	Adherence rate (%)	*N* (adherents/total)	Net increase (pp)	Relative increase (%)	Statistical significance
Baseline	11.2%	11/98	–	–	–
After phase 1 (education & counseling)	45.2%	28/62	+ 33.8	+ 302%	χ²=21.7, *p* < 0.001
After phase 2 (experience optimization)	75.0%	24/32	+ 29.8	+ 67% (from phase 1)	χ²=6.12, *p* = 0.013
Total improvement	–	–	+ 63.8	+ 569% (from the baseline)	*p* < 0.001

pp, percentage points.

**Table 3:** Comparison of secondary outcomes before and after the intervention.

Outcome measure	Before the intervention (baseline)	After the intervention (post-project)	Statistical significance
Knowledge mastery (%)	51.0	93.8	*p* < 0.01
Patient satisfaction (5-point Likert scale)	3.0	4.7	*p* < 0.01
Nasal symptom score (1–10 VAS)	8.3	3.2	*p* < 0.01
Weekly medication use (times/week)	5.2	2.1	*p* < 0.01

Data are presented as mean values. A lower Nasal Symptom Score indicates milder symptoms. VAS, visual analog scale.

## Discussion

This quality improvement study demonstrated that structured, patient-centered communication interventions can significantly improve patient adherence to physicians’ recommendations for nasal irrigation care. Traditionally, low adherence to nasal irrigation was thought to be an inherent deficiency of the patient, but our study suggests that it may be the result of insufficient communication between doctors and patients, and that adherence to nasal irrigation can be improved through specially designed health education and nasal irrigation teaching experiences.

After the first phase of The Multimodal Educational Strategy, patient adherence to nasal irrigation significantly increased (from 11.2% to 45.2%), highlighting the inadequacy of communication relying solely on verbal advice from physicians. The Multimodal Educational Strategy is an effective method to improve this. The success of our team’s strategy was attributed to its comprehensive learning style, which simultaneously addressed multiple learning barriers. The combination of visual aids, digital resources, and interactive one-on-one consultation teaching created a better learning experience^12^. This aligns with existing medical evidence, showing that patient education is most effective when it is interactive and personalized, and simultaneously equips patients with medical knowledge (“what the procedure of nasal irrigation is”) and self-efficacy (“I am confident I can perform nasal irrigation at home”)^10,13^. Physicians must also effectively play the role of a consultant, proactively helping to alleviate patients’ fears while building trust, which is a key factor in the initial success of improving nasal irrigation adherence.

Our research shows that health education is the most fundamental method of improvement. In the first phase, the Multimodal Educational Strategy increased nasal irrigation compliance to 45.2%, but more than half of rhinitis patients still did not perform nasal irrigation at home on their own. Therefore, we proposed a second-phase improvement plan. During the first phase, the research team collected feedback from patients and found that patients felt the traditional nasal irrigators were difficult to use and that the nasal rinse solution was too irritating. This patient feedback highlighted that rhinitis patients commonly experience ‘sensory defense,’ a reflexive fear of water entering or irritation in the nasal cavity, which hinders regular nasal irrigation. For example, outpatients frequently reported that using traditional nasal irrigation devices feels as uncomfortable as drowning, and standard saline is too irritating. The introduction of the Ergonomic Nasal Irrigation Device provided patients with a greater sense of control and security, directly alleviating anxiety associated with the sensation of drowning. Additionally, the Herbal Tea Rinse Saline Solution option transformed the nasal irrigation process from a tedious clinical chore into a more soothing and positive ritual. This shift from ‘enduring discomfort’ to ‘experiencing comfort’ is crucial. We directly addressed issues in the emotional and psychological action domains of compliance. This demonstrated that for daily self-administered nasal care routines, comfort, ease of use, and sensory experience are not trivial matters but core components of effective patient-centered communication and care. When the recommended behavior becomes easier and more enjoyable, patients are more likely to incorporate it into their lives. Most importantly, the second-phase intervention not only removed physical barriers but fundamentally reshaped the psychological relationship between patients and treatment. In the second phase of patient experience optimization measures, adherence to nasal irrigation further increased to 75.0%, rising from less than half of patients willing to perform nasal irrigation previously to three-quarters of patients willing to do so, which is of very significant clinical importance.

There are several limitations to this quality improvement study. First, it was conducted at a single medical center with patients from the routine outpatient clinic of one ENT doctor, and as a single-center project without a concurrent control group, it is susceptible to time trends and the Hawthorne effect. Additionally, it’s important to note that we used independent and different patient groups at each stage rather than a longitudinal design, so we cannot completely rule out confounding effects caused by time trends. Also, due to a limited number of nasal irrigators, the sample size in the second stage was relatively small (*n* = 32), so the specific results from this stage should be interpreted with more caution. Third, our findings rely on self-reported data from outpatients, which may introduce recall bias, and our follow-up period was relatively short, limiting our ability to assess long-term adherence. Finally, because the intervention was multimodal, we cannot statistically separate the individual contributions of any single measure (for example, using visual educational charts alone). Future studies should validate these results through multicenter randomized controlled trials and assess the effectiveness of each individual intervention, while also using digital health tools (like apps with personalized reminders) to support long-term self-management^14,15^.

This quality improvement study demonstrates that low adherence to nasal irrigation is not solely due to patient negligence; it is a predictable outcome caused by poor health communication quality and poor user experience. To improve clinical outcomes, we recommend that clinicians, health educators, and service managers shift from a passive ‘information provision’ model to an interactive consultation framework, proactively assessing and addressing individual patient barriers (such as fear and perceived difficulties) to build patients’ self-nasal irrigation capability. This approach requires formally training physicians and healthcare professionals to serve as key health consultants, supplemented with a standardized multimodal educational toolkit that combines visual aids, digital resources, and live demonstrations to accommodate diverse learning styles. Additionally, soliciting feedback and guiding patients to choose more comfortable, ergonomically appropriate options to prioritize patient experience are crucial, as this patient-centered approach is a powerful strategy to promote long-term adherence.

## Conclusions

Low adherence to nasal irrigation is not solely due to patients’ individual negligence, nor is it an insurmountable clinical problem. This is an issue that can be addressed through health education and effective doctor-patient communication. This study successfully demonstrated that a staged, patient-centered program that systematically addresses cognitive, emotional, and psychomotor barriers can significantly improve adherence. By replacing simple verbal instructions with a Multimodal Health Communication Strategy and refining the process based on patients’ communication feedback, we empowered patients to take an active role in their care. This not only improved knowledge retention and satisfaction but also yielded excellent clinical outcomes, providing a transferable model for improving self-management in other chronic diseases.

## Supplementary Information

Below is the link to the electronic supplementary material.

Supplementary Material 1

## Data Availability

The data presented in this study are available on reasonable request from the corresponding author.
